# The cost of aiming for the best answers: Inconsistent perception

**DOI:** 10.3389/fnint.2023.1118240

**Published:** 2023-04-03

**Authors:** Jeroen B. J. Smeets, Eli Brenner

**Affiliations:** Department of Human Movement Sciences, Vrije Universiteit Amsterdam, Amsterdam, Netherlands

**Keywords:** human, vision, haptics, Euclidean, illusion, space perception, multisensory, phenomenal space

## Abstract

The laws of physics and mathematics describe the world we live in as internally consistent. As these rules provide a very effective description, and our interaction with the world is also very effective, it seems self-evident that our perception follows these laws. As a result, when trying to explain imperfections in perception, we tend to impose consistency and introduce concepts such as deformations of visual space. In this review, we provide numerous examples that show that in many situations we perceive related attributes to have inconsistent values. We discuss how our tendency to assume consistency leads to erroneous conclusions on how we process sensory information. We propose that perception is not about creating a consistent internal representation of the outside world, but about answering specific questions about the outside world. As the information used to answer a question is specific for that question, this naturally leads to inconsistencies in perception and to an apparent dissociation between some perceptual judgments and related actions.

## 1. Introduction

Many textbooks and reviews start with the assumption that the goal of perceptual processes is to create a coherent or unified representation of the world (Hommel et al., 2001; Ernst and Bülthoff, 2004; Hagoort, 2005; Milner and Goodale, 2006; Dijkerman and de Haan, 2007). This representation is frequently described as being the most likely situation in the outside world to have caused the prevailing pattern of sensory stimulation (Kersten et al., 2004; Knill and Pouget, 2004; Fiser et al., 2010), suggesting that humans perform Bayesian inference to obtain this representation. We enjoy looking at images that are perceived to be globally incoherent while being locally coherent, such as many of the drawings by the Dutch graphical artist M. C. Escher. In such images, the inconsistency is not in the image itself, but arises because we interpret the image as representing a three-dimensional scene. Within that scene there are depicted depth relationships that cannot all be true, so it is evident that the depicted scene cannot exist (Penrose and Penrose, 1958). A problem with trying to create a coherent representation of this scene using Bayesian inference is that the likelihood of retinal stimulation being caused by a scene that cannot exist is zero; we will come back to this issue in the discussion. In the present paper we will review experimental results from the perceptual literature that are difficult to interpret by looking for a single coherent representation of the world that is consistent with the sensory stimulation. We will concentrate on situations that do not arise from the ambiguity of having to consider a dimension that is not in the stimulus, such as interpreting a painting as a three-dimensional scene.

Instead of assuming a coherent representation, we propose to assume that we ask ourselves questions about the world and remember the answers to these questions. This allows the answers to be inconsistent, i.e., there might not be a possible situation that gives rise to all these answers. The key to understanding how the situation is perceived is to carefully analyse which questions were asked during the prevailing task. Our approach in this review is to discuss which questions lead to inconsistent answers and discuss why that might be the case. Most of the questions that we will discuss are about different attributes that are related to each other by the physical and/or mathematical relations that govern the world around us. For instance, the velocity of an object is related to how its position changes, so we expect the answer to questions about how fast it is moving to be related to the answers to questions about where it is at various moments. However, because visual attributes are processed independently, the outcomes can be inconsistent (Smeets et al., 2002; Westheimer, 2008). We will start with a few cases in which only a single attribute is involved. In all cases, we will briefly discuss the sources of information that might be involved, without explaining all effects in detail.

## 2. Single-attribute inconsistencies

In this section, we discuss situations in which it is clear which attribute is questioned, but it is less clear to which item that attribute is related (the binding problem; Treisman, 1996). This often happens when that attribute differs between two scales (fine and coarse), such as when looking at an RGB monitor. Such a monitor consists of sets of three differently colored juxtaposed lights (red, green, and blue), whose brightness can be controlled independently. When observing the monitor from a very short distance, one sees the three colors of the lights, rather than the colors of the scene on the monitor. However, for normal viewing distances, the colors of the individual lights cannot be seen in isolation. They are mixed, so one perceives a whole range of colors in the scene. So, the answer to “which colors do you see on a RGB monitor?” depends on the viewing distance. Color perception contains an additional source of inconsistencies, because we use the name of a color (e.g., “yellow”) for three different properties: the wavelength of monochromatic light (i.e., 580 nm), the appearance of a light source that emits a combination of wavelengths (e.g., the color of the sun) or the reflective properties of a material (the color of rapeseed).

### 2.1. Brightness

**FIGURE 1:**
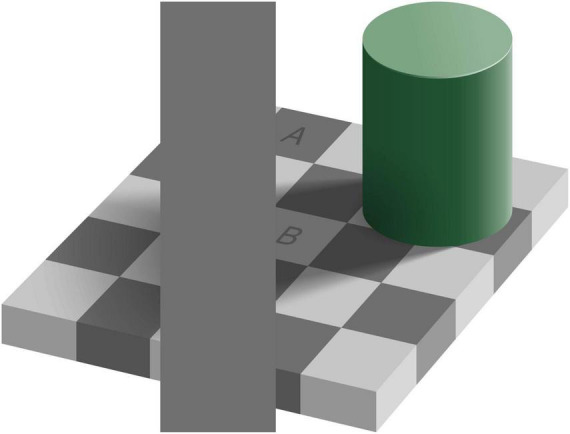
Inconsistent perception of brightness (original by Edward H. Adelson), CC BY-SA 4.0. Square A on the checkerboard appears to be darker than square B. At the same time, the vertical uniform gray rectangle that touches the squares is equally dark as A, as well as equally dark as B.

The easiest example of inconsistent perception to show in a paper is the inconsistency of brightness shown in Figure 1. When viewing the scene as a whole, square A seems darker than square B. However, when comparing the brightness of the squares with the occluding rectangle it is evident that they are all equally dark. These two answers seem inconsistent, until one realizes that “equally dark” is an answer to a question about the brightness of the pixels, whereas “A is darker than B” is an answer to a question about the reflectance properties of the surface (the “lightness” of the paint). The explanation is that in the perception of brightness of the whole scene, one not only considers the local luminance but also that of surrounding elements of the scene, as well as the organization of the scene (Adelson, 1993). In the example shown in the figure, there is a green cylinder on a checkerboard. With the light source being on the upper right, the cylinder casts a shadow on part of the checkerboard. Taking this shadow (or the luminance profile that it creates) into account, one perceives A as being darker than B. This line of thought answers the question about the reflectance of the depicted surfaces. In this example, the inconsistency is caused by asking two questions related to properties (the brightness of pixels versus the lightness of paint) that are only indirectly related according to physics: one needs to assume a light source to judge the lightness of the paint, but not to judge the brightness of a pixel. As the best answers to these questions rely on different information, an inconsistency can arise. In the rest of this review, we will generalize this idea to properties that are more tightly related according to physics.

### 2.2. Motion

**FIGURE 2:**
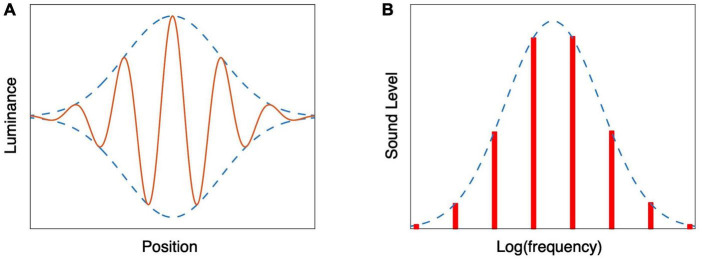
Schematic representation of stimuli consisting of a Gaussian envelope enclosing a finer structure. **(A)** A Gabor patch consists of a modulation of luminance by a sinusoid multiplied by a Gaussian. If the phase of the sinusoid is changing, the whole patch seems to move. **(B)** Shepard tones can be constructed by taking an infinite number of pure tones that are an octave apart and modulating the sound pressure level by a Gaussian.

We perceive motion by using specialized motion detectors (Barlow and Levick, 1965). Once detected, this motion has to be attributed (or bound) to the correct entity. Which entity it is bound to might depend on the question that you want to answer. An example of differences in attribution arises when a grating moves behind an aperture. If the aperture has sharp borders, we use the local motion to answer questions about motion of the grating, but not about motion of the aperture. If we blur the border, we also start using the motion signal to judge the motion of the aperture. Very strong blurring leads to a Gabor patch (Figure 2A). If the gaussian envelope is static and the grating moves rightward, the whole patch seems to move rightward. If the envelope is moving, and the grating moves with it but faster or slower, the whole patch is judged to move faster or slower (e.g., de la Malla et al., 2018). The effect of this motion is about 100%: 10 cm/s of relative motion adds about 10 cm/s to the perceived speed. One thus has inconsistent percepts of motion: when asked to judge the speed of the patch or the speed of the grating, one will report the same speed, but if one were to ask whether they move at the same speed, one would report that the grating is moving with respect to the patch. In addition to these inconsistencies due to misattribution of the motion signal, the motion is also inconsistent with the change in position, as we will discuss in section “3.1 Visual motion.”

### 2.3. Pitch and rhythm

Inconsistencies within a single attribute are not restricted to the visual domain. An auditory example of confusing local and global changes can be created using Shepard tones. A Shepard tone is a sound consisting of a superposition of pure tones whose frequencies are separated by an octave, resulting in an organ-like sound. If their sound pressure level is given by a Gaussian (Figure 2B), and the frequency of all the tones is increased by the same small fraction, the perceived pitch rises (Shepard, 1964). However, when asked to judge the timbre of the stimulus, the judgment will be based on the envelope, and thus will not change. The judgment of a change of pitch is based on a local analysis (the change in frequency of the individual tones during a short period of time), whereas the timbre is based on a more global analysis.

As judging the timbre of an individual tone is rather difficult, this inconsistency can be better illustrated by continuously increasing the frequency of the pure tones (and thus continuously perceiving an increase of the pitch). If we do so until all the pure tones have a one octave higher frequency, we will end up with exactly the same situation as at the start. We can thus create a sequence of tones of a continuously rising pitch (Supplementary Audio 2). This continuously rising pitch (with a stationary timbre) corresponds to the continuous rightward motion seen in stationary Gabor patches in which the grating is moving (section “2.2 Motion”). By moving the Gaussian envelope gradually to lower frequencies while increasing the frequencies of the pure tones, we keep the percept of a rising pitch (caused by the gradual shift of the pure tones), but now with a tone that is perceived as having a lower perceived pitch at the end (caused by the shift of the envelope; Risset, 1989).

One can also apply the trick of Figure 2B to the perception of rhythm: instead of the superposition of pure tones of a certain frequency, one creates a superposition of several simple rhythms that have repetition rates that differ by a factor of two (Risset, 1989). In this case, when the frequencies of the composing rhythms are gradually increasing (with their amplitudes given by the Gaussian envelope), the overall rhythm seems to accelerate continuously (Supplementary Audio 1). This percept is inconsistent in itself (a rhythm cannot continue to accelerate for a long period of time). If at any time a participant is asked to judge the rhythm at that moment, the answer will be the same. The percept of the change in rhythm is thus inconsistent with the lack of change in the percept of the rhythm.

## 3. Motion and position

According to physics, motion is the change in position per unit of time. Our perception of motion generally follows the change in position. However, there are various exceptions. For instance, Exner (1888) noted that the perception of motion of an object (e.g., the second hand of a clock) depends strongly on whether one directs one’s gaze at the object or is viewing it in the visual periphery. In contrast, one’s judgment of changing position (i.e., the time indicated by the second hand) is not affected. Even more extreme, in the very rare case of akinetopsia (Zeki, 1991), one cannot see motion despite seeing the changing position correctly. Without brain damage, one can have this experience when the scene is lighted by a stroboscope: you see a moving object at different places without perceiving motion. Motion is not only perceived visually, but also in the haptic and auditory domains.

### 3.1. Visual motion

Our perception of visual motion is partly based on retinal motion detectors (Barlow and Levick, 1965; van de Grind et al., 1986). Such detectors allow us to see motion of an object as a result of changes in the position of its image on the retina during a short period of time. In addition to this “retinal” motion, if we want to judge the object’s motion relative to our head or body (sometimes referred to as “egocentric” motion) we have to take the movements of our eyes into account (Braun and Schütz, 2022). One of the ways to do so is by considering the scene, and thus relative motion (Duncker, 1929; Brenner and van den Berg, 1996), in a similar way as we take the scene into account to perceive brightness (section “2.1 Brightness”). A simple reaction time study has shown that absolute and relative motion are processed independently (Smeets and Brenner, 1994). The optimal way to combine the three different sources of motion information (retinal slip of the target’s image, relative retinal motion, extra-retinal information) depends on the question, leading to many inconsistencies.

One of the best-known motion illusions is the motion after-effect or waterfall illusion. If one looks at a waterfall for some time, and then shifts one’s gaze to an adjacent rock, this rock appears to be moving upward. As this after-effect can last for tens of seconds (Hershenson, 1989), a coherent percept would entail perceiving the rock at a totally different location after some time. This is not the case: the motion after-effect is “a sensation of motion without displacement” (Anstis et al., 1998, p. 111), which is a clear example of inconsistency.

Souman et al. (2006) performed an experiment to distinguish between retinal motion and egocentric motion. Participants were pursuing a horizontally moving dot in the dark and were asked to judge the motion of a second, vertically moving dot. They had to judge either the motion direction, the position where it appeared on the screen or the position where it disappeared. They found that the perceived direction of motion was inconsistent with the direction between the perceived locations of the positions at which it appeared and disappeared. The perceived direction of motion was closer to the retinal motion than one would predict from the perceived positions.

As we mentioned at the beginning of this section, our percept of motion is influenced by motion of the background (Duncker, 1929). You can measure this by letting participants compare moving stimuli with and without a moving background. In a study where we used a stimulus in which the perceived velocity of the target was influenced considerably by the background velocity (Smeets and Brenner, 1995) we also asked the participants to indicate the target’s position after 500 ms of motion. The effect of background motion on the judged position was clearly smaller than one would expect on the basis of 500 ms of the biased motion percept. In another experiment, the inconsistency between reports of motion and position change were even more pronounced (Brenner and Smeets, 1997). In that experiment, background motion to the left made the target appear to move rightward but its perceived position ended up being further to the left.

Another example of perception of motion that is inconsistent with the change in perceived positions is the finding that the perceived speed of a moving stimulus such as a rotating wheel depends on the visual characteristics of the stimulus. The perceived speed of a rotating wheel is reduced if the spatial frequency is high (Campbell and Maffei, 1979) and the luminance contrast is very low (Campbell and Maffei, 1979; Cavanagh et al., 1984). Both manipulations do not affect the perceived position of any spoke of the wheel, so the change of perceived position is not affected by these manipulations. There are many videos on the internet that show such reductions of perceived speed that leave perception of the changing position unaffected. For a combination of spatial frequency and background motion, an example can be found in Brenner and Smeets (2022, Movie 5).

A non-visual way to induce a percept of visual motion is the oculo-gyral illusion (Graybiel and Hupp, 1946; Carriot et al., 2011). This illusion occurs if a person’s body rotates (mildly accelerating) in complete darkness together with a small light rotating at the same velocity as the body. One’s impression is that the light is moving in the direction of the body’s rotation, but importantly for our argument, one perceives the position of the light to remain constant, which is inconsistent with the simultaneously perceived motion. Irrespective of the detailed mechanisms causing this illusion (Carriot et al., 2011), it can be regarded as an example of participants answering a different question than the experimenter poses. Although it is evident from the laws of physics that one could define motion relative to various reference frames (e.g., one’s own body or the outside world), humans have a single motion percept that cannot be modified by an assessment of one’s own motion (Brenner, 1991). In a similar way as it is very difficult to report the color of pixels rather than the color of paint when asked about color (or brightness, Figure 1), it is very difficult to report motion relative to a specific non-visual reference frame, rather than one’s default motion percept.

In section “2.2 Motion” we already discussed the motion illusion that is elicited if the grating in a Gabor patch moves relative to the Gaussian envelope. This motion not only produces a very strong illusion of motion, but also a very subtle illusory position shift (De Valois and De Valois, 1991; de la Malla et al., 2019). Most importantly, the illusions of motion and position are not only inconsistent in size, but also caused by different mechanisms (Linares and Holcombe, 2008).

**FIGURE 3:**
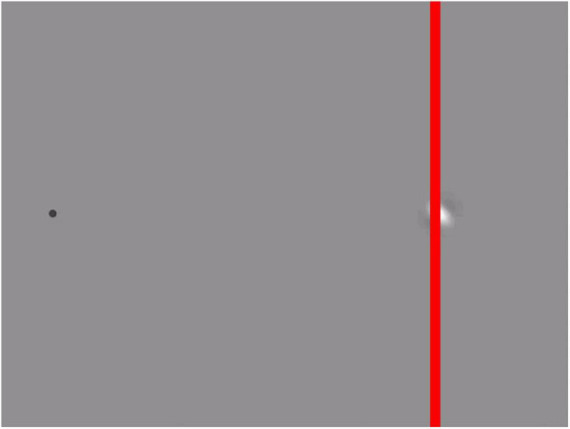
Inconsistency in the double drift illusion (see Supplementary Video 1). When fixating the dot on the left, the Gabor patch appears to move vertically, parallel to the red line. Despite continuing to move vertically, the Gabor appears to cross the red line when it is about halfway along its path. The movie is based on the stimulus used by Lisi and Cavanagh (2015), but with an additional vertical red line that reveals the inconsistency: a change in perceived relative position that is inconsistent with the perceived direction of motion.

If the grating’s motion is perpendicular to the direction of motion of the envelope, the illusion becomes an illusion of motion direction, sometimes referred to as the “double-drift illusion” (Lisi and Cavanagh, 2015, 2017). This illusion works best when viewed using peripheral vision. It makes a vertically moving patch appear to be moving diagonally and can make a diagonally moving patch appear to move vertically. When you add a vertical line to such a stimulus (Figure 3), the inconsistency becomes visible: although the patch seems to move vertically, parallel to the red line, it appears to do so to the left of the red line in the upper half of the image, and to the right of the vertical line in the lower half.

### 3.2. Localization of flashes in the presence of visual motion

Nijhawan (1994) reported that when a bar was flashed next to a moving bar while participants were fixating, the participants saw the flash lagging the moving object. He interpreted this as evidence that the eye and brain extrapolate the trajectory of the moving bar, so sampling the internal representation at the time of the flash yields this percept. This finding has had a tremendous influence on psychology, leading to claims that humans are predicting the present (Cavanagh, 1997). This claim is based on the idea that our percept of the difference in location of the flash and the moving bar correspond to a single frame of a consistent internal representation. By introducing unpredictable changes to the target motion such as a change of speed (Brenner and Smeets, 2000) or direction (Eagleman and Sejnowski, 2000; Whitney et al., 2000), various authors have shown that the perceived lagging of the flash cannot be due to motion extrapolation, but must be due to asynchronous sampling of positions (for an alternative view, see Hubbard, 2014; Hogendoorn, 2020). This is a similar deviation from the idea of a consistent internal representation as the spatial examples that we have discussed so far, where related spatial attributes did not match across time when asking different questions, but in the temporal domain.

Our explanation for the systematic error is that there is an asynchronous sampling of positions. Instead of judging the difference in position from a snapshot of the internal representation (which is some consistent percept), participants answer the implicit question which is “where is the target at the moment of the flash.” They can only start to localize the moving target when the visual system has detected the flash. This detection takes time. Assuming that it takes 60 ms, this reasoning explains the experimental results. So, the percept is based on incoherent information: it combines the location of the flash with that of the bar 60 ms later. If participants use this strategy to localize objects at the moment of an event, the “flash-lag” effect should also be present if the flash is replaced by a very brief auditory or tactile stimulus. This is indeed the case (Alais and Burr, 2003; Cellini et al., 2016). In line with the explanation of sampling, presenting a noise burst together with the flash reduced the flash lag effect and its variability (Vroomen and de Gelder, 2004).

### 3.3. Localization around the moment of a saccade

The timing issues that we discussed in the previous paragraphs also play a role when localizing objects while the eye is moving, leading to substantial mislocalizations [reviewed by Schlag and Schlag-Rey (2002)]. Many authors have found that flashes presented around the moment of a saccade are mislocalized. As the reported positions are closer to where one is fixating, these mislocalizations have been described as a “compression of visual space” (Ross et al., 1997; Lappe et al., 2000). Based on such a compression of space, one would predict that when the flashes are presented on a structured background, this background would be compressed as well, so participants would report the correct location relative to the background. We tested this experimentally, by presenting flashes on a background consisting of red and green parts. This added background did not affect the compression of the location of the flashes, so that targets that were flashed on a green part of the background were readily localized on the red part of the background. If this mislocalization would have been due to a consistent compression of visual space, the background should have been compressed as well, so they would have seen a flash on a green background. However, the percept was inconsistent with this prediction: participants indicated that they saw the flash on a red background (Maij et al., 2011a). In a similar fashion, when participants were asked about the size of a flash that was presented near a saccade, they show strongly compressed localization, whereas the perceived object size was not compressed (Matsumiya and Uchikawa, 2001; Luo et al., 2010). These systematic errors in localization around the moment of a movement are not specific for eye movements, but also occur for tactile localization with a moving hand (Maij et al., 2011b,^2013^).

### 3.4. Motion and position in haptics

We perceive the position and motion of our limbs on the basis of efferent and proprioceptive afferent signals. In general, this yields a consistent kinesthetic percept. This percept can be perturbed by vibration of a muscle tendon, leading to effects that seem to correspond to stretching the muscle. If the muscle is an elbow flexor, the percept is a combination of feeling that the arm is moving in the extension direction and feeling that it is more extended. An inconsistency arises when the vibration continues: the arm feels as if it is continuously extending, but the angular judgment remains at a constant (erroneous) value. This is a similar inconsistency as we discussed for the motion aftereffect. Apparently, kinesthetic position and motion are based on different information. This has been confirmed in an experiment by Sittig et al. (1987). They studied how the perceptual errors depended on the frequency at which the tendon was vibrated and found that the perturbation increased with the vibration frequency. However, this increase saturated at different frequencies for the judgment of the limb’s position and velocity. Having established tendon vibration as a tool to dissociate between position and velocity, Sittig et al. (1987) could use tendon vibration to determine to what extent the control of goal-directed movement relied on position or velocity. Their conclusion was that it depended on the speed of the movement: slow movements relied on position information, whereas fast movements relied on velocity information.

## 4. Spatial relations

### 4.1. Visual size and position

**FIGURE 4:**
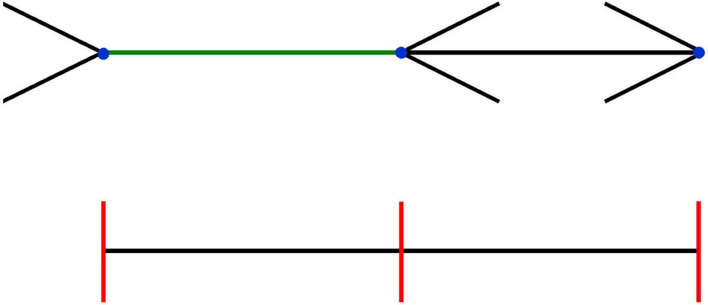
Inconsistency between size and position in the Brentano version of the Müller-Lyer illusion. The three red lines are (and appear to be) equidistant, and are clearly aligned with the blue dots, which are thus also equidistant. Nevertheless, the green line connecting the two dots on the left seems longer than the black line connecting the dots on the right. When the orientation of the arrows is flipped (see Supplementary Video 2), the length of the green line appears to change, while the blue dots seem to remain static.

The inconsistency between size and position is easier to illustrate than the inconsistencies that involve motion that we discussed in the previous section. We created an example in Figure 4, in which it is clear that the Brentano version of the Müller-Lyer illusion affects perceived size (green line segment), but not the perceived positions of the endpoints of the same line (blue dots). This figure is inspired by various studies in which participants were not only asked to indicate the perceived size of a line segment, but also the perceived locations of the endpoints (Gillam and Chambers, 1985; Mack et al., 1985). From our previous arguments it should be clear that we agree with the authors of the studies that inspired this figure that the reason the percept of size (the green line appears longer than the black one) is inconsistent with that of position (blue dots appear aligned with the equidistant red lines) is that one is asking a different question, that relies on different information. If one is asked to determine a position, one tends to fixate it with one’s eyes, so one can judge its position using (extra-retinal) information about eye orientation. If one is asked to judge size (green line), the answer is based on interpreting the retinal image size.

If size and position were consistent, one might be tempted to explain the Müller-Lyer illusion as being caused by averaging information near the endpoint, leading to shifts in perceived positions of the endpoints (Bulatov, 2017). If this would be a valid explanation, one would expect that inverting the direction of the fins on one side would shift both endpoints in the same direction. Indeed, an equivalent position illusion exists in which the location of the center of the bar appears to be shifted (the Judd illusion). However, the strength of the size illusion (the Müller-Lyer illusion) does not correspond with the strength of the position illusion (Judd illusion) that you obtain when inverting one of the fins (Stuart et al., 1984). The inconsistency between size and position is also present in various other size illusions. We have demonstrated this inconsistency in a combination of the Sander and the Oppel–Kundt illusion (Smeets and Brenner, 2019). However, some size illusions affect perceived positions as well. A notable example is the Ebbinghaus illusion, for which the effect of the flankers on the perceived position of two opposite sides corresponded with the effect on perceived size (Smeets and Brenner, 2019). This illusion does not induce an inconsistency between the perceived size and positions. Why the flankers influence the perceived location of the edges of the central disk is not clear.

Various studies have used variants of the Müller-Lyer illusion in goal-directed movements and tried to manipulate to what extent information about size was used. They reasoned that if you make a movement from one end of the illusion to the other end, the perceived size of the connecting line is useful information about the distance that needs to be moved, and will therefore be used to make the movement. On the other hand, if the movement starts outside the illusion, the perceived size of the line is of little relevance, and will therefore not be used to make the movement. A size illusion should only influence a movement if size is used to plan or guide the movement. Indeed, it has been reported for saccades (de Grave et al., 2006), stylus pointing movements (de Grave et al., 2004), and beanbag throwing (Canal-Bruland et al., 2013) that movements between the vertices of the illusion are affected, but movements that start outside the illusion are not. For the pointing movements, the authors varied whether the hand and target were visible during the movement, and found that the more was visible during movement execution, the more participants relied on egocentric position information rather than allocentric size information (de Grave et al., 2004).

This inconsistency between size and position is conceptually not limited to the visual domain. It should hold also for the haptic domain. In analogy to the finding that the strength of a visual size illusion depends on the extent to which perceptual judgments rely on egocentric and allocentric information, the inconsistency here will depend on the extent to which participants rely on instantaneous tactile information or combine tactile information with proprioception. Indeed, it has been reported that exploration strategies influence the strength of haptic geometrical illusions (Gentaz and Hatwell, 2004).

### 4.2. Visual space

**FIGURE 5:**
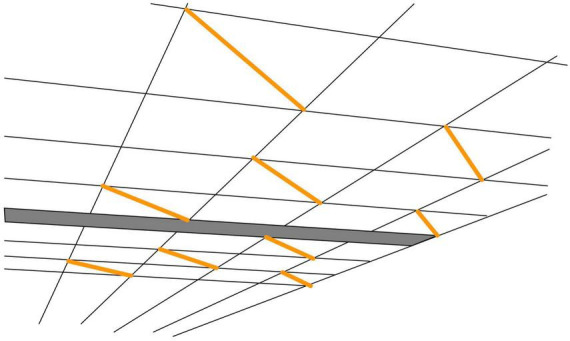
Inconsistency in perspective viewing. Drawing based on the photograph in Kappers and te Pas (2001). Despite seeing a ceiling consisting of aligned square tiles and seeing that the orange lines are diagonals of such square tiles, the orange lines do not appear to be parallel. The figure obviously shows a two-dimensional rendition; Kappers and te Pas reported that the effect was even stronger in an actual room: the physically parallel fluorescent tubes appeared visually extremely non-parallel.

One of the concepts that are frequently used in the perception literature is “visual space,” “a coherent self-organized dynamic complex that is structured into objects, backgrounds, and the self” (Indow, 1991). Illusions are then considered as distortions or deformations of this visual space (Gregory, 1963). A nice example of such a deformation was noted by Kappers and te Pas (2001), when viewing a ceiling consisting of square tiles. On some of the tiles, a fluorescent tube was mounted along the diagonal, so all tubes are parallel to each other (Figure 5). Despite the fact that the squares look like squares, the tubes don’t seem to be parallel to each other. This is inconsistent with the rules of Euclidian geometry.

A distortion of visual space can be thought of as a deformation such that the rules of geometry that we learn at high school are not valid anymore. In more formal terminology: after the deformation, visual space is non-Euclidian. For instance, we have learnt at school that the angles of an equilateral triangle are 60^°^. However, in a deformed space, the angles of an equilateral triangle need not be 60^°^. A 2D example of such a deformed space (a deformed plane) is the surface of the earth. When you start at the equator and move to the north pole in a straight line and then make a 90^°^ right turn at the north pole, you will move back toward the equator. If you make a second 90^°^ right turn once you reach the equator, you will move along the equator back to your original position. Each of the three paths will be 1/4 of the circumference of the sphere, so your movement path was an equilateral triangle, but the angles are all 90^°^. The fact that the red lines in Figure 4 appear equidistant and appear to be aligned with the blue dots that do not appear equidistant is only inconsistent if one assumes Euclidean geometry.

There are many papers discussing the possibility that visual space is non-Euclidean (Luneburg, 1950; Foley, 1972; Wagner, 1985; Cuijpers et al., 2003) or even is not metric at all (Todd et al., 2001; Koenderink et al., 2002; Wagner, 2008). All these studies have argued that visual space is nevertheless consistent, i.e., that optical points and lines exist and that two points define a unique line, and two lines a unique (intersection) point (Koenderink et al., 2002). For instance, Todd et al. (2001) showed, using a bisection task, that participants’ perceptual space was distorted, but the judgments were consistent (i.e., had an affine structure). However, when performing bisections to judge the center of a square formed by two Judd-figures, we found a clear violation of the affine structure: the extent to which the Judd-illusion affected the judgment depended on the strategy used to make the judgment: the order of the questions that were asked (Smeets et al., 2009). A consistent but non-Euclidean space cannot explain the fact that when switching the orientations of the arrows in Figure 4, the length of the green line-segment changes, but the blue dots remain at the same positions.

### 4.3. Location and direction

**FIGURE 6:**
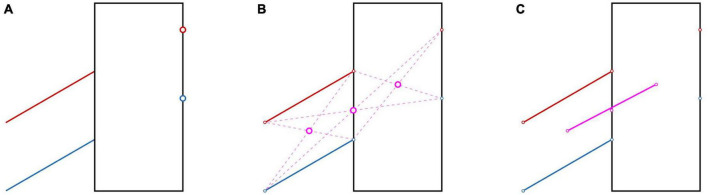
How the Poggendorff illusion warps visual space. **(A)** The red and blue disks on the right are perceptually aligned with the lines with the same color on the left. The alignment is illusory, as they are not aligned. **(B)** The three purple disks are the intersections between the diagonals obtained by connecting each of the three red points on the upper line with the two blue points on the lower line that are farthest away. **(C)** If the manipulations in **(A,B)** would have resulted in a homogeneous space, the three purple disks should be aligned. The straight line shows they do not: the central disk is slightly below the straight line. This is obviously a consequence of the error we introduced in **(A)**. We showed experimentally that if one constructs the three positions indicated by the purple disks perceptually, they are also not aligned (Smeets et al., 2009).

In the Poggendorff illusion (Figure 6A), one misjudges the alignment between oblique lines and points due to perceptual errors in extrapolating the lines and in judging their orientations (Ninio and O’Regan, 1999). Can we describe these misjudgments with a deformation of space? As this illusion is concerned with collinearity, one can test this using a property of collinearity of three points that holds for any space (e.g., Euclidean, affine or projective), regardless of whether it is metric (Pappus hexagon theorem; Koenderink et al., 2002). We tested this theorem by asking participants to extrapolate two lines in a Poggendorff illusion by moving a cursor on a laptop screen. Subsequently they were asked to use these two points and the endpoints of the line to indicate the equivalents of the three purple disks in Figure 6B. Finally, we removed the central of these three points, and asked the participants to place a point halfway between the two remaining points. The latter point was slightly above the point that we removed (similar to the physical misalignment illustrated in Figure 6C), leading to the conclusion that Pappus’s hexagon theorem does not hold (Smeets et al., 2009). So, visual space does not only violate the rules of Eucledian geometry and the more relaxed rules of affine geometry (based on the results on the Judd figures discussed in section “4.2 Visual space”), but it also violates the even more relaxed rules of projective geometry. The reason for this violation of the rules of even the most relaxed geometry is that the illusion affects perception of collinearity without affecting the localization of the items that are involved. The take-home message of this result is that there is no consistent visual space.

### 4.4. Haptic space

Haptics is the combination of tactile and proprioceptive information. We already discussed in the last sentence of section “3.3 Localization around the moment of a saccade” that this combination can give inconsistencies when the hand is moving. But also in a static situation, inconsistencies can arise. For instance, when rotating a bar until it feels parallel to another bar, participants make systematic errors, both when the bars are on a horizontal plane (Kappers, 1999) and when they are on a frontoparallel plane (Hermens et al., 2006). The systematic errors depend on the question that is asked: they were clearly different when participants (in a separate experiment) reported the orientation of individual bars by reporting the clock-time it represented (Hermens et al., 2006; Kappers, 2018) rather than whether the bars were parallel. These findings can be explained by assuming that participants use a combination of egocentric and allocentric information to judge orientation, and that the relative weight of these sources of information depends on the question that is asked (Kappers, 2007).

A second example of inconsistencies can be found in temporal order judgments. It seems self-evident that when both hands of a participant are stimulated, participants are able to judge which of the two hands is stimulated first. Indeed, participants can do this reliably, even with intervals as short as 70 ms (Yamamoto and Kitazawa, 2001). Surprisingly, their performance deteriorates when the hands are crossed: for some intervals they even report the reverse order. This is not a simple confusion between left and right, as a similar misattribution of the first stimulus can occur between hand and foot (Badde et al., 2019). If both hands are moving in such an experiment in which participants misjudge the temporal order and participants are asked to indicate where the hand was touched first, they do not indicate where the first touch was felt, but a location close to where the other hand was at the moment of the touch (Maij et al., 2020). The authors concluded that humans construct external touch locations post-hoc and on demand, in line with the flash-lag effect that we discussed in section “3.2 Localization of flashes in the presence of visual motion.”

**FIGURE 7:**
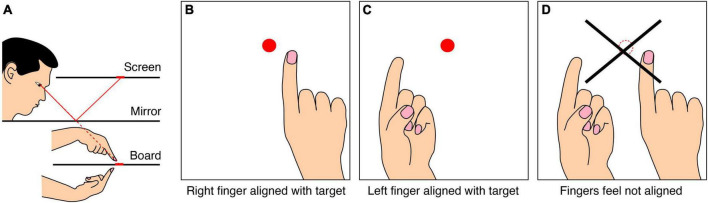
Schematic representation of an experiment showing an inconsistency in sensory alignment of the tips of invisible fingers. **(A)** The set-up used (Kuling et al., 2017). When the right index-finger feels as if it is at the location of a visual target **(B)**, and the left finger feels as if it is at the same visual location **(C)**, the two fingers in general do not feel aligned **(D)**.

A last example of the inconsistency of perception of space is an experiment in which participants are asked to match three locations: that of the invisible left index-finger, the invisible right index-finger and a visual target. When moving an unseen index-finger to the location of a continuously visible target (Figure 7A), one will make some systematic error. This error differs between the two fingers (Figures 7B, C). If one would have a consistent internal representation of space, one would expect that if one would place both fingers at the respective positions that perceptually match the same visual target, one would feel the fingers at the same location (Figure 7D). This is not the case (Kuling et al., 2017). The reason for this inconsistency is that different questions were asked. The tasks in Figures 7B, C require a transformation from a proprioceptive reference frame of the hand to a visual reference frame in which the target is presented. The errors that are present in that transformation are not present in a direct comparison of the two proprioceptive locations (Figure 7D).

### 4.5. Associations

In the above, we discussed situations in which our percept is inconsistent with the laws that govern our world. We explained some of the inconsistencies by information helping some judgments but not others. Most of the information sources reflect the lawful regularities in the world. In addition to these regularities that reflect the laws of physics, there are also regularities that are not lawful but based on a statistical evaluation. For instance, large objects are generally heavier and have surfaces that are less curved than small objects. One might expect that such associations would influence perception, so that for instance having a more strongly curved surface would make an object appear smaller. However, when tested experimentally, the opposite pattern is observed: feeling that an object’s surface is strongly curved makes it feel larger (Plaisier and Ernst, 2013).

A very robust illusion that goes beyond spatial relationships is based on the experience that large objects are in general heavier than small objects: increasing the size of an object makes it feel lighter (the size-weight illusion, see for instance Charpentier, 1891; Ross, 1966; Plaisier and Smeets, 2012). A similar effect is also found for the material of which the object is made. If an object appears to be made of a high-density material, it will feel lighter (the material-weight illusion; Ellis and Lederman, 1999). An interesting suggestion is that these weight illusions are due to the fact that participants do not answer the posed question about the object’s weight, but a question about a property that is related to the weight: the density of the material (Peters et al., 2018; Wolf et al., 2018). Although there is some experimental evidence that seems to be in conflict with this explanation (Plaisier and Smeets, 2015), the notion of answering a question related to density can explain various findings on the size-weight and material-weight illusion. For instance, the finding that these illusions are not related to the forces used to lift the object (Flanagan and Beltzner, 2000; Buckingham et al., 2009; Platkiewicz and Hayward, 2014) becomes less surprising if the illusion arises because the participants are not really answering a question about the weight.

## 5. Discussion

In the above, we provided many examples in which perceptual judgments about related properties are clearly inconsistent with each other, without most scientists noting this. The question we have reserved for this last section is whether the assumption of consistency has led to serious misconceptions. We think that it has, especially in the interpretation of apparent inconsistencies between perception and action as providing support for functional specialization of the dorsal and ventral visual stream (Goodale and Milner, 1992; Milner and Goodale, 1993, 2006). Our interpretation of such apparent inconsistencies between perception and action is the same as our interpretation of the perceptual inconsistencies that we have discussed above. In both cases, they are the consequence of asking slightly different questions. In the following paragraphs, we will discuss these issues on the basis of a few examples.

We will start by an individual with visual form agnosia (patient DF) who was not able to make judgments about shape and orientation but nevertheless could accurately reach out and grasp a pencil orientated at different angles (Milner et al., 1991). The action capabilities of DF were quantified as the relation between the orientation of a card and that of a slot when posting a card, and between peak grip aperture and object size when grasping (Goodale et al., 1991). If one assumes that these tasks rely on slot orientation and object size, these findings would be very remarkable. However, card posting does not rely on a visual estimate of object orientation (Hesse et al., 2021) and maximum grip aperture does not rely on a visual estimate of object size (Schot et al., 2017; Smeets et al., 2020). Both motor acts rely on the processing of egocentric location information–using the fact that two locations specify size and orientation of an object–rather than relying on judgments of those attributes themselves (Schot et al., 2017; Smeets et al., 2020; Hesse et al., 2021).

In analogy with the inconsistency between perceptual judgments, one would also expect inconsistencies in aspects of an action. These are indeed present, for instance when reaching to pick up an object. If the object appears larger due to a size illusion, one expects it to be heavier, and thus uses more force to grasp and lift the object. Experiments on the Ponzo illusion indeed revealed this effect (Brenner and Smeets, 1996; Jackson and Shaw, 2000). If one would have a consistent internal representation underlying this action, one would expect that one would grasp the apparently larger object with a larger peak grip aperture. This is not the case as peak grip aperture during grasping is insensitive to the Ponzo illusion that influences the forces during the same reach-to-grasp movements (Brenner and Smeets, 1996; Jackson and Shaw, 2000). In a similar way, a speed illusion can affect how fast you move toward a moving target, without affecting where you initially aim during this movement (Smeets and Brenner, 1995).

One subfield of neuroscience that has been heavily influenced by the doctrine of a single consistent representation is multisensory integration. In this subfield, the idea is that “To perceive the external environment our brain uses multiple sources of sensory information derived from several different modalities, including vision, touch and audition. All these different sources of information have to be efficiently merged to form a coherent and robust percept” (Ernst and Bülthoff, 2004, p. 162). To achieve this coherent percept, one generally assumes that the various sources of information are combined in a statistically optimal fashion, both within and across modalities (Ernst and Banks, 2002; Hillis et al., 2002). The combination is optimal in that the weights given to the various signals when averaging them is the one that will give the highest possible precision of the combined estimate. In experiments, the weight is usually determined by using stimuli in which cues specify different values of an attribute, for instance slant. When evaluating slant in a three-dimensional scene, the optimal weights to give to binocular and monocular cues depends on the viewing distance and the size of the slanted surface, because the precision is limited by the density of the receptors in the stimulated part of the retina and the relationship between distance and vergence angle. As predicted by this view, the weights assigned to such cues vary across viewing conditions and stimulus properties (Backus et al., 1999). Most importantly, this view predicts that the weights are completely independent of the task.

Is the weighting of the cues indeed independent of the task? We have some clear evidence that the weights can be task dependent. A first example of such task dependency is the finding that weights are not determined by the retinal stimulation, but by how this scene is segregated into objects (Muller et al., 2009a). Participants in that study were asked to align two transparent surfaces that were both slanted around the same axis at the same place. These surfaces were patterned differently, so that the relative reliability of monocular and binocular cues differed. When introducing the same cue conflict between monocular and binocular cues for slant, the participants could have aligned the surfaces by simply matching the binocular slant and the monocular slant. However, they did not do so, but first estimated the slant of each of the two surfaces (using different weights), and then aligning these surfaces. The weighting of cues thus depended on the task (which surface to judge), rather than on the retinal location. An even clearer demonstration of the influence of a task on the weights given to cues is that repeatedly experiencing that one’s judgment of the slant of a cue-conflict stimulus is biased can make one adjust the weights to reduce this bias (van Beers et al., 2011). Both experiments show that the way one combines sensory information is not only determined by their precision, but is also influenced by specific task-dependent issues: in these examples segregation into surfaces and feedback.

In the above discussion of slant perception, the monocular cue for slant refers to the deformation of the assumed actual shape in the retinal image. For instance, since the retinal image of a slanted rectangle is a trapezoid, a trapezoid on the retina could indicate that one is looking at a slanted rectangle. Whether one should interpret a trapezoid on the retina as such, and how confident one should be about this interpretation, should depend on how likely one considers it to be that the object in the scene is either a rectangle or a trapezoid. Surprisingly, one tends to ignore visual evidence about the actual shape of objects in a scene when judging the reliability of monocular slant cues (Muller et al., 2009b). However, there are situations in which one’s assumptions about the shape can be modified in a way that influences one’s percept. For instance, van Ee et al. (2002) reported that presenting large conflicts between monocular and binocular slant cues can give rise to bistable percepts, whereby one switches between accepting and rejecting the assumption that the object is rectangular. Moreover, they report that one can voluntarily switch between these two percepts, which corresponds to the observer switching between the questions “What is the slant of the rectangle?” and “What is the slant of the trapezoid?”.

**FIGURE 8:**
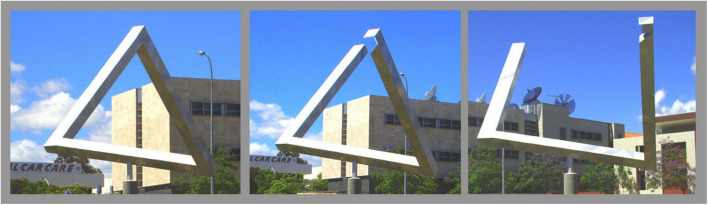
A sculpture by Brian MacKay and Ahmad Abas in East Perth, Western Australia from three different viewpoints. In the left image, the structure looks like a Penrose triangle. One perceives an impossible structure, despite the existence of a real structure that yields the same retinal stimulation. This figure is based on pictures by Bjørn Christian Tørrissen (CC BY-SA 3.0).

At the beginning of the introduction we mentioned the Penrose triangle (Figure 1 in Penrose and Penrose, 1958), which is, according to the caption, a “Perspective drawing of impossible structure.” However, the caption is misleading: it is a drawing that is perceived as an impossible structure. There are several structures that give the same retinal stimulation as the impossible figure one perceives (an example in Figure 8). In all these structures, the misperception of the whole as being impossible is due to the violation of expectations about the parts. For the example in Figure 8, one expects that the perfect fit on the top of the left image is caused by two connected bars, rather than by a right bar with a peculiarly shaped end that is designed to visually match the left bar when viewed from this point. Using Bayesian methods to answer questions about the parts leads to a non-Bayesian percept of the whole image. This nicely reflects our reasoning in that striving to answer specific questions in the best possible manner will not guarantee consistency between the answers since different measures and assumptions are informative for different judgments, and informative measures might be given different weights for different judgments.

In this review, we have provided abundant evidence for inconsistencies in how we make perceptual judgments and use spatial information to guide our movements. We cannot explain why one has the impression of having a coherent internal representation. This impression is so strong that many prefer to assume two consistent internal representations (one for judgments and one for the control of action) over giving up the idea of consistency. A possible reason is that we are generally not confronted with inconsistencies, as perception consists of answering questions one-by-one. In a similar fashion, we normally do not realize that we have a blind spot or are color blind in the visual periphery (O’Regan and Noë, 2001): as soon as we ask questions, we move our eyes, and get the requested information. Considering perception in terms of sequentially asking the questions that are most relevant at that instant is not only a way to describe our perception. It is can also be used to describe our natural visuomotor behavior in terms of using a series of visual routines that extract relevant information from the optical array (Ballard et al., 2000).

## Author contributions

JS wrote the first draft and created the figures. Both authors contributed to the conception of the review, subsequent versions of the manuscript, read, and approved the final version.
